# Insiders as outsiders: burnout and its determinants among acupuncture practitioners in China’s public hospitals

**DOI:** 10.3389/fpubh.2026.1765792

**Published:** 2026-02-19

**Authors:** Jian Dong, Qihui Zhou, Chanyuan Long, Yihui Feng, Yingruo Wang

**Affiliations:** 1 School of Grammar and Law, Shandong University of Science and Technology, Qingdao, China; 2 Shandong Engineering Research Center for Tissue Rehabilitation Materials and Devices, School of Rehabilitation Sciences and Engineering, University of Health and Rehabilitation Sciences, Qingdao, China; 3 Dongbei University of Finance and Economics, Dalian, China

**Keywords:** acupuncture practitioners, burnout, burnout-related factors, China, public hospitals

## Abstract

**Background:**

With the growing global interest in health protection and traditional medicine, acupuncture practitioners are receiving widespread international recognition. However, research on burnout among this professional group remains limited—particularly in the context of public hospitals. This study adopts a novel perspective that bridges acupuncture practitioners and occupational health, aiming to assess both burnout level and its determinants among acupuncture practitioners in public hospitals. It helps reveal the unique occupational stressors and health risks faced by this group within institutional medical settings.

**Methods:**

Drawing on the person-environment fit theory, a survey was conducted among 614 acupuncture practitioners employed in China’s public hospitals, by using the Chinese version of the Maslach Burnout Inventory–Human Services Survey (MBI-HSS). The study used SPSS for statistical analyses, including one-way ANOVA, Pearson correlation, and multiple linear regression, to examine the main factors contributing to acupuncture practitioners’ burnout.

**Results:**

The findings indicated a moderate level of burnout among acupuncture practitioners (mean = 2.63 ± 0.89) in China’s public hospitals, with emotional exhaustion (2.69 ± 0.90), depersonalization (2.56 ± 0.97), and reduced personal accomplishment (2.61 ± 0.93) as the primary dimensions. Key determinants were categorized into four levels: individual factors (gender, marital status, age, years of work experience, professional title); organizational factors (institutional support, organizational management systems, remuneration, career advancement, departmental competition, interpersonal relationships); occupational factors (work intensity, work specialization); and societal factors (patient negativity, public misunderstanding).

**Conclusion:**

Burnout is prevalent among acupuncture practitioners in China’s public hospitals and is influenced by multiple factors. Alleviating burnout in this context requires a multi-level strategy: developing a human-centered occupational support system, aligning organizational management with the specific needs of acupuncture practice, implementing tailored and sustainable work mechanisms, and fostering a socially supportive environment. This study broadens the scope of burnout research by applying it to acupuncture practitioners and provides actionable recommendations to improve working conditions and promote the well-being of acupuncture practitioners in public healthcare systems.

## 1 Introduction

As global awareness of health and well-being increases, acupuncture has achieved widespread international recognition and is now practiced in 196 countries and regions (1, 2). As core professionals, acupuncture practitioners have also flourished worldwide, emerging as an essential occupational group in contemporary global healthcare systems. In China, public hospitals are the main providers of acupuncture services, where acupuncture practitioners are critical to health promotion, disease prevention, and rehabilitation (3), often regarded as “harmonizers of human health.” However, this rapid professional expansion is accompanied by significant challenges. Acupuncture practitioners in public hospitals frequently contend with heavy workloads, high-pressure environments, and sustained psychological strain, all of which elevate their risks of burnout. Burnout not only endangers their own mental and physical well-being, but also compromises the quality of patient care, potentially leading to medical errors and strained therapeutic relationships (4). Given these risks, it is essential to understand and address burnout among this group.

Despite burnout being widely studied in the medical field, studies focusing specifically on acupuncture practitioners within public hospitals remain scarce. To address this gap, our analysis focuses on acupuncture practitioners working in China’s public hospitals and employs a burnout scale specifically tailored to this professional group to comprehensively examine their current status of burnout. Furthermore, guided by Person-Environment Fit Theory, we propose a novel four-dimensional analytical framework encompassing ‘personal-organizational-occupational-societal’ factors to identify the key determinants of burnout systematically. Our study not only contributes to broadening the scope of burnout research and enriching the theoretical understandings of occupational health research on acupuncture practitioners but also offers actionable insights for improving public hospital management and advancing the quality of acupuncture services.

### 1.1 Burnout

Burnout is a psychological syndrome stemming from prolonged work-related stress, manifesting as a state of profound physical and mental exhaustion. Burnout was first introduced by Freudenberger (5) in his work with staff at free clinics (5). Maslach et al. later conceptualized burnout into a three-dimensional model: emotional exhaustion, depersonalization, and reduced personal accomplishment. Emotional exhaustion reflects the feeling of being emotionally overextended and drained by one’s work. Depersonalization involves a cynical, detached, and impersonal attitude toward those receiving care. Reduced personal accomplishment is characterized by the tendency to evaluate one’s work negatively, accompanied by feelings of diminished competence and achievement (6). Extensive research has linked burnout to severe adverse outcomes, including depression, sleep disorders, substance misuse, and a decline in physical health (7). For healthcare professionals, including acupuncture practitioners, burnout poses a dual threat: it severely jeopardizes their personal well-being and critically undermines the quality and safety of patient care (8, 9). Therefore, burnout represents a significant risk to the integrity of the healthcare systems within which they operate.

### 1.2 Acupuncture practitioners’ burnout in public hospitals

Acupuncture stands as one of the world’s oldest and most enduring medical therapies (10). By stimulating specific acupoints, acupuncture regulates the flow of qi and blood, aiding in disease prevention, chronic condition management, rehabilitation, and overall health maintenance (11). Today, it is globally recognized as an effective, low-cost treatment with minimal side effects (1). In China, public hospitals form the cornerstone of the healthcare system and serve as the primary providers of acupuncture services. As key specialists in acupuncture, acupuncture practitioners within public hospitals possess a solid foundation in classical theories, such as meridian pathways and acupoint functions (12). They are highly proficient in various techniques and bear the critical responsibilities of ensuring both the safety and efficacy of clinical treatments. However, systemic challenges within public hospitals, including overwhelming workloads, limited opportunities for career advancement, and increasingly tense doctor-patient relationships, place significant physical and psychological strain on acupuncture practitioners (13). Consequently, they face heightened risks of burnout.

Burnout often manifests as chronic physical exhaustion, emotional fatigue, diminished motivation, and a reduced sense of engagement with patient care (14). Over time, burnout can degrade the quality of treatment and increase the likelihood of medical errors and conflicts with patients (9). Given these, targeted research on burnout among acupuncture practitioners is urgently needed.

### 1.3 Factors influencing burnout among acupuncture practitioners in public hospitals

As a population with a high prevalence of burnout, acupuncture practitioners in public hospitals experience burnout shaped by a variety of factors. More specifically, from a clinical perspective, each patient session requires accurate syndrome differentiation, precise acupoint selection, and meticulous needle application (15, 16). This process demands that acupuncture practitioners command a vast body of complex and abstract theoretical knowledge, including the theories of acupoints, yin and yang, and qi. Meanwhile, this process demands that acupuncture practitioners maintain constant mental focus to adapt treatments in response to subtle changes in a patient’s condition (17). These prolonged cognitive and emotional demands can place acupuncture practitioners in sustained tension, significantly heightening their risk of fatigue and burnout (18). From an organizational perspective, systemic inefficiencies within public hospitals exacerbate this stress. Practitioners frequently contend with inadequate departmental support, rigid administrative hierarchies, limited opportunities for career advancement, unequal resource distribution, and poor collegial or interdepartmental relationships (19, 20). These organizational factors collectively undermine practitioners’ job satisfaction and profoundly impact their psychological and physical well-being (21). From a societal perspective, a lack of widespread public understanding of acupuncture’s principles (22), which stems from insufficient health education and cultural misconceptions, can foster patients’ skepticism or mistrust (23). This further strains the practitioner-patient relationship and exacerbates burnout among acupuncture practitioners. Investigating the factors that influence burnout among acupuncture practitioners is critical. It not only deepens the theoretical understanding of occupational health for acupuncture practitioners, but also provides essential evidence base for developing targeted preventions to alleviate burnout.

### 1.4 Current study

Previous research has firmly underscored the unique and indispensable role of acupuncture practitioners within the modern healthcare system. However, within public hospitals, which are the main providers of medical services, acupuncture practitioners suffer from different levels of burnout, which in turn leads to various social concerns. Although some research has touched upon the occupational health of acupuncture practitioners, insufficient focus has been given to the issue of acupuncture practitioners’ burnout within public hospital settings. In particular, there is a lack of a coherent analytical framework to examine the complex relationships between multiple influencing factors and acupuncture practitioners’ burnout. Consequently, several key questions remain unanswered: What is the true severity of burnout among acupuncture practitioners in public hospitals? What are its primary drivers? And how can acupuncture practitioners’ burnout be alleviated through the optimization of hospital management systems? This study is designed to address these questions directly.

To this end, we employ a validated burnout scale, tailored to acupuncture practitioners, to assess the prevalence of burnout among practitioners in public hospitals. Guided by Person-Environment Fit Theory, we construct a novel four-dimensional analytical framework encompassing individual, organizational, professional, and societal factors. Within this framework, we advance the following hypotheses: (a)Individual factors, including gender, marital status, age, years of work experience, education level, and professional title, significantly influence acupuncture practitioners’ burnout. (b)Organizational factors, including institutional supports, organizational management systems, remuneration, career advancement, departmental competition, and interpersonal relationships, are key determinants of burnout. (c)Occupational factors, including work intensity, work specialization, work environment, use of modern equipment, and occupational hazards, directly contribute to burnout. (d)Societal factors, including patient negativity and public misunderstanding, further exacerbate practitioners’ burnout. Based on this analytical framework, we will employ one-way analysis of variance (ANOVA) to identify the individual factors influencing acupuncture practitioners’ burnout, aiming to explore the impact of individual characteristics (such as gender, marital status, age, years of work experience, education level, and professional title) on burnout. Additionally, we will employ correlation analysis and regression analysis to identify the organizational, occupational, and societal factors influencing acupuncture practitioners’ burnout, aiming to examine the relationships between these factors and occupational burnout. Building on this, we further investigate how these factors contribute to the onset of acupuncture practitioners’ burnout.

By investigating burnout and its influencing factors among acupuncture practitioners in China’s public hospital, this paper aims to: (a) deeply understand acupuncture practitioners’ current burnout status; (b) identify influencing factors of burnout; and(c) propose strategies to alleviate the negative impacts of burnout. Ultimately, this paper seeks to help acupuncture practitioners enhance their ability to cope with high-pressure work environments in public hospitals. Furthermore, this paper seeks to raise public awareness of acupuncture practitioners’ real life in public hospitals, urge greater supports for this critical workforce, advocate for optimization of public hospital management, and foster a more sustainable and supportive work environment for acupuncture practitioners’ occupational health, thereby contributing to the improvement of acupuncture services quality and the sustainable development of the acupuncture industry.

## 2 Methods

### 2.1 Participants and procedure

This study aimed: (1) to evaluate the current prevalence of burnout among acupuncture practitioners in public hospitals and (2) to identify and analyze the key influencing factors. To meet these objectives, we developed a comprehensive questionnaire designed to measure acupuncture practitioners’ burnout status and its influencing factors in a public hospital. The target population was acupuncture practitioners employed in public hospitals across China. To ensure a nationally representative sample, no geographic restrictions were applied. Data were collected over 2 months primarily via the WeChat platform, where a survey link was distributed to eligible participants through professional networks. A total of 760 responses were submitted. Following a rigorous data screening process that included logic verification and manual review to ensure quality, 614 responses were validated for analysis, yielding an effective response rate of 80.8%. To enhance the instrument’s clarity and relevance, a preliminary version of the questionnaire was reviewed by acupuncture practitioners, and their feedback was incorporated into the final design. All participants volunteered anonymously and were fully informed of the study’s purpose and procedures prior to participation. All identifying information was removed to protect confidentiality. The study protocol was reviewed and approved by the Ethics Committee of China University of Health and Rehabilitation Sciences, and all procedures were conducted in strict accordance with institutional ethical guidelines.

### 2.2 Measures

#### 2.2.1 Measurement of demographic characteristics

Our study examined six demographic variables: gender, marital status, age, years of work experience, education level, and professional title. Gender was considered a basic demographic variable because it is a core individual trait and may influence acupuncture practitioners’ work attitudes and decision-making behaviors. Marital status was considered because it is an indicator of acupuncture practitioners’ living status and may influence their work responsibility and job stability. Age was considered for its association with acupuncture practitioners’ professional cognition and life attitude. Years of work experience were considered for their association with acupuncture practitioners’ career experience and professional maturity. Education level was considered for its association with acupuncture practitioners’ theoretical training and Knowledge attainment. Professional title was considered for its association with acupuncture practitioners’ professional seniority and work autonomy. Due to their close relevance to individual characteristics, these demographic variables were categorized as individual factors potentially influencing acupuncture practitioners’ burnout. We explore how these individual factors contribute to burnout among acupuncture practitioners using questionnaire data collection and analysis.

#### 2.2.2 Burnout scale

MBI-HSS is one of the most widely used tools for measuring occupational burnout (24). However, to accurately assess the true state of burnout among acupuncture practitioners in China’s Public Hospitals, it is necessary to further integrate the specific context of China’s public hospitals and the unique work characteristics of acupuncture practitioners into MBI-HSS. This will enhance the applicability of MBI-HSS within acupuncture practitioners in China’s Public Hospitals. Therefore, based on the theoretical framework of the MBI-HSS scale and in consideration of the unique occupational characteristics of acupuncture practitioners in Chinese public hospitals, we made appropriate adjustments to the original MBI-HSS. We revised certain items of MBI-HSS to better fit Chinese public hospital’s context, so as to make the findings more interpretable and persuasive. This resulted in the creation of “Burnout Questionnaire for Acupuncture Practitioners in China’s Public Hospitals”. The revised scale consists of 22 items categorized into three core dimensions: Emotional Exhaustion (Items 7–15), capturing the degree of physical and emotional fatigue experienced; Depersonalization (Items 16–20), reflecting a sense of detachment or indifference toward patients and the public; and Reduced Personal Accomplishment (Items 21–28), assessing feelings of diminished professional competence and job-related fulfillment. All items were rated on a five-point Likert scale ranging from 1 (never) to 5 (always), with higher composite scores indicating more severe levels of burnout.

#### 2.2.3 Burnout influencing factors

The Person-Environment (P-E) Fit Theory served as the foundational framework for identifying factors contributing to acupuncture practitioners’ burnout. This theory posits that individual behavior arises from the interaction between personal characteristics and environmental conditions (25). A strong alignment between the individual and their environment promotes well-being, job satisfaction, and adaptability, thereby reducing burnout risk. In contrast, a poor fit generates psychological strain and increases susceptibility to burnout (26). Acupuncture practitioners’ burnout are influenced not only by individual factors but also by external environmental factors such as organizational conditions, occupational demands, and societal perceptions. There is a strong dependency between acupuncture practitioners and their work environment. The application of the P-E fit to analyze the factors influencing acupuncture practitioners’ burnout has significant theoretical advantages and practical relevance. This theory provides an effective framework for analyzing the multiple influencing factors of acupuncture practitioners’ burnout, offering valuable insights into causes of burnout, and designing targeted interventions. P-E fit is commonly conceptualized across multiple dimensions, including person-organization, person-job, person-group, person–person, and person-vocation alignment (27). Guided by this theoretical lens and accounting for the specific professional context of acupuncture practitioners in public hospitals, this study organizes influencing factors of burnout into four dimensions: ‘individual-organizational-occupational-societal’ factors. A structured questionnaire was developed to operationalize and measure variables within these four domains (see Appendix 1).

##### 2.2.3.1 Individual factors

Individual factors comprise six demographic variables: gender, marital status, age, years of work experience, education level, and professional title. These variables are measured through Questions 1–6 in the questionnaire.

##### 2.2.3.2 Organizational factors

This dimension examines how organization-related factors in public hospital lead to acupuncture practitioners’ burnout. Prior studies suggest that inadequate institutional support given by public hospitals for acupuncture departments often leads to perceived marginalization, which in turn undermines acupuncture practitioners’ professional identity (28, 29). Rigid management systems and burdensome procedures in public hospitals make acupuncture practitioners feel constrained and restricted, thereby heightening their job dissatisfaction (30). The low remuneration of acupuncture practitioners in public hospitals leads them to perceive a mismatch between their efforts and rewards, thereby generating a sense of unfairness (31). The considerable challenges in career advancement and the limited opportunities for professional development leave acupuncture practitioners uncertain about their career prospects, thereby undermining their work motivation (32). Acupuncture departments hold a weak position in public hospital competition, leading acupuncture practitioners to experience professional frustration. The complex interpersonal relationships within public hospitals often subject acupuncture practitioners to internal exhaustion, resulting in feelings of depression and irritability (33). Based on these findings, organizational factors primarily encompass institutional supports, organizational management systems, remuneration, career advancement, departmental competition, and interpersonal relationships, which are reflected in Questions 29–34 of the survey.

##### 2.2.3.3 Occupational factors

This dimension examines how work-related factors in public hospitals contribute to the burnout of acupuncture practitioners. Prior studies suggest that acupuncture practitioners often face high patient volumes and prolonged acupuncture procedures, leading to physical and mental fatigue. The abstract nature of acupuncture theory and the high specialization required in clinical practice impose heavy learning demands and intensive clinical pressures on acupuncture practitioners (34), thereby leading to tension and anxiety. Crowded and noisy work environments could distract acupuncture practitioners, intensify mental fatigue, and provoke negative emotions (29). The use of modern rehabilitation equipment erodes the traditional skills and clinical expertise of acupuncture practitioners, weakens their professional security, and provokes anxiety and concern (35). Additionally, acupuncture practitioners often face occupational hazards such as moxa smoke (36, 37), repetitive manual operations, and poor postures (38), leading to chronic occupational illnesses. Based on these findings, occupational factors primarily encompass work intensity, work specialization, work environment, use of modern equipment, and occupational hazards, which are reflected in Questions 35–39 of the survey.

##### 2.2.3.4 Societal factors

This dimension examines how social actors, such as patients and the general public, lead to acupuncture practitioners’ burnout. Prior studies suggest that one significant source of acupuncture practitioners’ burnout arises from patients who, due to illness-related distress, treatment discomfort, or unmet recovery expectations, may express negative emotions (30, 39). Such interactions can escalate tensions within the therapeutic relationship, leading to emotional exhaustion and depersonalization among acupuncture practitioners. Furthermore, insufficient public awareness and understanding of acupuncture (40)—often stemming from cultural biases (9), a preference for western medicine, or general skepticism toward traditional practices—can result in a lack of professional respect and recognition. These societal perceptions directly undermine acupuncture practitioners’ professional identity and diminish their sense of personal accomplishment. Based on these findings, societal factors primarily encompass patient negativity and public misunderstanding, which are reflected in Questions 40–41 of the survey.

### 2.3 Data analysis

Data analysis was conducted using IBM SPSS Statistics 29.0, employing both descriptive and inferential statistical methods, with a significance threshold set at *p* < 0.05. First, descriptive statistics were used to compute means and standard deviations (SD) for the overall burnout scale and its three subscales to assess the general level of burnout among acupuncture practitioners in a public hospital. Next, one-way analysis of variance (ANOVA) were employed to examine whether burnout levels differed significantly across various individual factors. Subsequently, correlation analysis was conducted to evaluate the relationships between the overall burnout scale and the three categories of influencing factors: organizational, occupational, and societal factors. Finally, multiple linear regression analysis was performed with overall burnout as the dependent variable and organizational, occupational, and societal factors (including their subdimensions) as independent variables to examine the effects of these factors on acupuncture practitioners’ burnout within a public hospital.

## 3 Results

### 3.1 Demographic information for samples

The study included 614 acupuncture practitioners from public hospitals. The sample consisted of 417 males (67.9%) and 197 females (32.1%). Regarding marital status, 425 participants were married (69.2%), while 171 (27.9%) were single, and 18 (2.9%) were divorced or widowed. Age distribution was relatively balanced: 136 participants (22.1%) were aged 18–30, 194 (31.6%) were 31–40, 147 (23.9%) were 41–50, and 137 (22.4%) were over 50 years. Work experience varied: 145 participants (23.6%) had fewer than 5 years of experience, 166 (27.0%) had 6–10 years, 162 (26.4%) had 11–20 years, and 141 (23.0%) had more than 20 years. In terms of education level, 254 participants(41.4%) hold bachelor’s degrees, 248 (40.4%) master’s degrees, and 84 (13.7%) doctoral degrees; 28 (4.5%) had less than an associate degree, highlighting the high educational profile of this profession within China’s public hospitals. Professional titles were distributed as follows: 215 participants (35.0%) held junior titles, 289 (47.1%) intermediate titles, 64 (10.4%) senior titles, and 46 (7.5%) had no designated title. In summary, our study reveals that acupuncture practitioners in public hospitals are predominantly male, mostly married, and exhibited a balanced age distribution. They are generally well-educated and experienced, though hold comparatively lower professional titles.

### 3.2 Prevalence of burnout among acupuncture practitioners in public hospital

#### 3.2.1 Current status of burnout

Burnout was measured using a five-point Likert scale, with higher scores indicating greater severity. Based on one-third of total scores, burnout levels were categorized into three groups: low (<2.0), moderate (2.0–4.0), and high (>4.0). As shown in Table 1, the mean overall burnout score among acupuncture practitioners was 2.63 (SD = 0.89), indicating a moderate level of burnout. The scores for the three subdimensions were also in the moderate range: emotional exhaustion (2.69 ± 0.90), depersonalization (2.56 ± 0.97), and reduced personal accomplishment (2.61 ± 0.93). Further analysis revealed that 68.7% of acupuncture practitioners (*n* = 422) experienced moderate burnout, while 6.5% (*n* = 40) experienced high burnout, and 24.8% (*n* = 152) experienced low burnout. Among the subdimensions, emotional exhaustion had the highest proportion of practitioners in the moderate category (*n* = 416), followed by reduced personal accomplishment (*n* = 408) and depersonalization (*n* = 393). These results indicate that moderate burnout is prevalent among acupuncture practitioners in public hospitals.

**Table 1:** Number of burnout in various dimensions.

Dimensionality	High burnout		Moderate burnout		Low burnout		Mean burnout	Std. deviation
	Number of people	Percentage	Number of people	Percentage	Number of people	Percentage		
Emotional exhaustion	48	7.8%	416	70%	136	22.2%	2.69	0.9
Depersonalization	58	9.4%	393	64%	163	26.6%	2.56	0.97
Reduced personal Accomplishment	49	8%	408	66.4%	157	25.6%	2.61	0.93
Overall burnout	40	6.5%	422	68.7%	152	24.8%	2.63	0.89

#### 3.2.2 Relationship between individual characteristics and burnout

This study examined the impact of demographic differences on burnout, providing valuable insights into how individual factors contribute to burnout among acupuncture practitioners. We used one-way analysis of variance (ANOVA), obtaining the following key results (Table 2). The results indicate that (1) Gender was significantly associated with acupuncture practitioners’ burnout, especially for overall burnout (*p* = 0.018 < 0.05), depersonalization (*p* < 0.001), and reduced personal accomplishment (*p* = 0.009 < 0.05). Male practitioners exhibited higher burnout levels (2.72 ± 0.90) than females (2.65 ± 0.92). (2) Marital status was significantly associated with overall burnout (*p* = 0.015 < 0.05), emotional exhaustion (*p* = 0.025 < 0.05), depersonalization (*p* = 0.011 < 0.05), and reduced personal accomplishment (*p* = 0.029 < 0.05), among all groups, married practitioners exhibited the highest burnout levels (2.75 ± 0.89). (3) Age was significantly associated with overall burnout (*p* = 0.007 < 0.05), depersonalization (*p* < 0.001), and reduced personal accomplishment (*p* = 0.002 < 0.05), burnout among acupuncture practitioners tends to increase with age. (4) Years of work experience was significantly associated with overall burnout (*p* < 0.001), emotional exhaustion (*p* = 0.009 < 0.05), depersonalization (*p* < 0.001), and reduced personal accomplishment (*p* < 0.001), burnout among acupuncture practitioners shows an overall upward trend with increasing years of work experience. (5) Educational level showed no significant association with any burnout dimension (all *p* > 0.05), it does not lead to significant differences in burnout among acupuncture practitioners. (6) Professional title was significantly associated with overall burnout(*p* = 0.014 < 0.05), emotional exhaustion(*p* = 0.015 < 0.05), depersonalization (*p* = 0.014 < 0.05), and reduced personal accomplishment (*p* = 0.04 < 0.05), among all groups, acupuncture practitioners with intermediate professional title exhibited the highest level of occupational burnout (2.75 ± 0.82). These findings suggest that gender, marital status, age, years of work experience, and professional title are salient factors influencing burnout among acupuncture practitioners, while education level does not demonstrate a significant effect.

**Table 2:** Relationship between acupuncture practitioners’ individual characteristics and burnout.

Individual characteristics	Emotional exhaustion			Depersonalization			Reduced personal accomplishment			Overall burnout		
	Mean burnout	Std. deviation	*p*-value	Mean burnout	Std. deviation	*p*-value	Mean burnout	Std. deviation	P-value	Mean burnout	Std. deviation	*p*-value
Gender												
Male	2.72	0.9	0.0.378	2.67	0.94	<0.001	2.68	0.91	0.009	2.69	0.87	0.018
Female	2.65	0.92		2.33	1		2.47	0.96		2.51	0.9	
Marital status												
Married	2.75	0.89	0.025	2.63	0.96	0.011	2.67	0.93	0.029	2.7	0.87	0.015
Divorced/Widowed	2.4	1.03		2.11	0.97		2.21	1.03		2.27	0.97	
Single	2.57	0.91		2.43	0.98		2.5	0.91		2.51	0.89	
Age												
18–30	2.48	0.89	0.096	2.33	0.97	<0.001	2.42	0.94	0.002	2.53	0.86	0.007
31–40	2.68	0.85		2.47	0.96		2.55	0.88		2.56	0.91	
41–50	2.67	0.92		2.46	0.96		2.48	0.91		2.61	0.9	
>51	2.77	0.82		2.78	0.87		2.78	0.88		2.86	0.0.83	
Years of work experience												
<5 years	2.49	0.88	0.009	2.34	0.95	<0.001	2.43	0.89	<0.001	2.44	0.86	<0.001
6–10 years	2.73	0.94		2.59	1.04		2.61	0.97		2.65	0.94	
11–20 years	2.7	0.91		2.46	0.94		2.52	0.89		2.58	0.86	
>20 years	2.85	0.85		2.87	0.88		2.89	0.91		2.87	0.83	
Education level												
Associate degree or below	2.49	0.88	0.623	2.34	0.95	0.267	2.43	0.89	0.846	2.44	0.86	0.672
Bachelor	2.73	0.94		2.59	1.04		2.61	0.97		2.65	0.94	
Master	2.7	0.91		2.46	0.94		2.52	0.89		2.58	0.86	
Doctor	2.85	0.85		2.87	0.88		2.89	0.91		2.87	0.83	
Professional title												
Undecided	2.49	0.88	0.015	2.37	1.07	0.014	2.44	0.94	0.04	2.45	0.91	0.014
Junior	2.6	0.94		2.48	1		2.53	0.96		2.55	0.92	
Intermediate	2.81	0.85		2.96	0.9		2.72	0.87		2.75	0.82	
Senior	2.6	0.97		2.38	1.06		2.47	1.05		2.5	0.96	

### 3.3 Factors affecting burnout among acupuncture practitioners in public hospital

#### 3.3.1 Correlation analysis

Following the exploration of the relationship between individual factors and acupuncture practitioners’ burnout, a bivariate correlation analysis was further conducted to explore relationships between ‘organizational-occupational-societal’ factors and acupuncture practitioners’ burnout. The data distribution was tested, and the results indicated that the data approximately follow a normal distribution. Therefore, pearson correlation analysis was employed, with the total burnout score as a single composite measure,to further explore the relationship between the total burnout among acupuncture practitioners and 13 influencing factors across organizational, occupational, and societal domains. As summarized in Table 3: (1) All organizational factors demonstrated significant positive correlations with practitioners’ burnout: institutional supports (*r* = 0.712**, *p* < 0.001), organizational management systems (*r* = 0.693**, *p* < 0.001), remuneration (*r* = 0.684**, *p* < 0.001),career advancement (*r* = 0.684**, *p* < 0.001), departmental competition (*r* = 0.743**, *p* < 0.001), and interpersonal relationships (*r* = 0.714**, *p* < 0.001). (2) All occupational factors demonstrated significant positive correlations with practitioners’ burnout: work intensity (*r* = 0.717**, *p* < 0.001), work specialization (*r* = 0.729**, p < 0.001), work environment (*r* = 0.396**, *p* < 0.001), use of modern equipment (*r* = 0.402**, *p* < 0.001), and occupational hazards (*r* = 0.353**, *p* < 0.001). (3) All societal factors demonstrated significant positive correlations with practitioners’ burnout: patient negativity (*r* = 0.428**, *p* < 0.001), public misunderstanding (*r* = 0.450**, *p* < 0.001). These results indicate that all 13 factors across ‘organizational-occupational-societal’ dimensions are significantly associated with acupuncture practitioners’ burnout. Among these, departmental competition showed the strongest correlation, while occupational hazards showed the weakest correlation.

**Table 3:** Correlation analysis of burnout and its factors.

Factors affecting burnout	Correlation coefficient	Sig. (bilaterally)
Organizational factors		
Institutional supports	0.712**	*p* < 0.001
Organizational management systems	0.693**	*p* < 0.001
Remuneration	0.684**	*p* < 0.001
Career advancement	0.684**	*p* < 0.001
Departmental competition	0.743**	*p* < 0.001
Interpersonal relationships	0.714**	*p* < 0.001
Occupational factors		
Work intensity	0.717**	*p* < 0.001
Work specialization	0.729**	*p* < 0.001
Work environment	0.396**	*p* < 0.001
Use of modern equipment	0.402**	*p* < 0.001
Occupational hazards	0.353**	*p* < 0.001
Societal factors		
Patient negativity	0.428**	*p* < 0.001
Public misunderstanding	0.450**	*p* < 0.001

#### 3.3.2 Multiple linear regression analysis

A multiple linear regression analysis was conducted to further investigate the impact of organizational, professional, and societal factors on the burnout of acupuncture practitioners in public hospitals. The total burnout score was entered as the dependent variable, and the 13 related factors in ‘organizational-occupational-societal’ dimensions were entered as independent variables. As presented in Table 4, the adjusted R-squared value was 0.669, indicating that the model explained 66.9% of the variance in burnout. The adjusted R-squared value exceeds 50%, demonstrating strong explanatory power. The Durbin-Watson statistic of 1.859, close to the ideal value of 2, indicated no significant autocorrelation and satisfied the model’s independence assumption. The overall regression model was statistically significant (*p* < 0.001), confirming that at least one of the independent variables significantly contributed to burnout. A further analysis of the standardized coefficients revealed that organizational factors (*β* = 0.818, *p* < 0.001), occupational factors (*β* = 0.661, *p* < 0.001), and societal factors (*β* = 0.472, *p* < 0.001) exerted significant positive effects on burnout among acupuncture practitioners in a public hospital.

**Table 4:** Multiple linear regression analysis of burnout among acupuncture practitioners.

Model	Non-standardized coefficient		Standard coefficient	*t*-value	Sig.	R square	Adjusted R square	Durbin-Watson	F
	B	Standard error	Beta						
(Constant)	0.678	0.065		10.483	<0.001	0.67	0.669	1.859	
Organizational factor	0.705	0.02	0.818	35.132	<0.001				413.664
Occupational factor	0.636	0.029	0.661	21.784	<0.001				*p* < 0.001
Societal factor	0.384	0.029	0.472	13.251	<0.001				

^a^Dependent variable: total score of burnout.

To further identify the influence of specific factors within each independent variable on acupuncture practitioners’ burnout, we conducted multiple linear regression analysis for the detailed factor indicators (Table 5): For organizational factors, burnout was significantly associated with multiple factors: institutional support (*β* = 0.176, *p* < 0.001), organizational management systems (*β* = 0.103, *p* = 0.009), remuneration (*β* = 0.079, *p* = 0.042), career advancement (*β* = 0.110, *p* = 0.004), departmental competition (*β* = 0.286, p < 0.001), and interpersonal relationships (*β* = 0.197, *p* < 0.001). These results indicate that limited institutional support, rigid management systems, inadequate remuneration, difficult career advancement, strained departmental competition, and complex interpersonal relationships are all associated with higher levels of burnout among acupuncture practitioners in public hospitals. For occupational factors, burnout was significantly associated with multiple factors: work intensity (*β* = 0.395, *p* < 0.001) and work specialization (*β* = 0.437, *p* < 0.001). These results indicate that high work intensity and stringent work specialization requirements are both associated with higher levels of burnout among acupuncture practitioners in public hospitals. For societal factors, burnout was significantly associated with multiple factors: patient negativity (*β* = 0.163, *p* < 0.001) and public misunderstanding (*β* = 0.221, *p* < 0.001). These results indicate that greater transmission of negative emotions from patients and stronger cognitive bias of the general public toward acupuncture are both associated with higher levels of burnout among acupuncture practitioners in public hospitals.

**Table 5:** Multiple linear regression analysis of factors influencing burnout.

Factors affecting burnout	Unstandardized coefficients		Standardized coefficients	t	Sig.	Collinearity statistics	
	B	Std. error	Beta			Tolerance	VIF
Organizational factors							
Institutional supports	0.131	0.029	0.176	4.542	<0.001	0.354	2.826
Organizational management systems	0.075	0.029	0.103	2.615	0.009	0.342	2.92
Remuneration	0.059	0.029	0.079	2.039	0.042	0.352	2.843
Career advancement	0.081	0.028	0.11	2.896	0.004	0.37	2.704
Departmental competition	0.216	0.29	0.286	7.555	<0.001	0.371	2.697
Interpersonal relationships	0.147	0.028	0.197	5.235	<0.001	0.374	2.672
Occupational factors							
Work intensity	0.293	0.028	0.395	10.638	<0.001	0.463	2.16
Work specialization	0.333	0.028	0.437	11.823	<0.001	0.467	2.143
Work environment	0.027	0.028	0.035	0.939	0.348	0.471	2.124
Use of modern equipment	0.004	0.028	0.005	0.147	0.883	0.473	2.113
Occupational hazards	−0.013	0.026	−0.017	−0.493	0.622	0.529	1.889
Societal factors							
Patient negativity	0.163	0.04	0.215	4.121	<0.001	0.469	2.134
Public misunderstanding	0.221	0.039	0.293	5.63	<0.001	0.469	2.134

^a^Dependent variable: total score of burnout.

## 4 Discussion

Based on a novel perspective of the cross-integration of acupuncture practitioners and occupational burnout, our study offers a comprehensive analysis of the burnout status of acupuncture practitioners in China’s public hospitals. Furthermore, we identify various influencing factors of acupuncture practitioners’ burnout from ‘individual-organizational-occupational-societal’ perspectives, and explore the impact of these factors on burnout. The findings indicate that 68.9% of acupuncture practitioners experience moderate burnout, with notable differences across three dimensions: emotional exhaustion, depersonalization, and reduced personal accomplishment. Through one-way ANOVA, we find that, within individual factors, gender, marital status, age, years of work experience, and professional title significantly affect acupuncture practitioners’ burnout in China’s public hospitals. Through multiple regression analyses, key factors influencing acupuncture practitioners’ burnout are identified, including organizational factors (institutional support, organizational management systems, remuneration, career advancement, departmental competition, and interpersonal relationships), occupational factors (work intensity and work specialization), and societal factors (patient negativity and public misunderstanding). This study enriches the current research on burnout among acupuncture practitioners and provides some significant insights for optimizing the practice environment of acupuncture practitioners and alleviating their burnout.

Analysis of individual factors reveals that, firstly, gender is a key factor in the burnout of acupuncture practitioners, which aligns with earlier research (41). Our study further found that male practitioners were more prone to higher levels of burnout compared to their female counterparts in China’s public hospitals. The possible reason is that men generally exhibit weaker empathetic capacities than women (42). Consequently, male practitioners are less likely to receive positive emotional feedback from patients during clinical interactions, making them more vulnerable to emotional exhaustion and depersonalization. Secondly, marital status was a key factor in acupuncture practitioners’ burnout, which is in line with earlier research (43). Our study further found that the level of burnout among married acupuncture practitioners was higher than that of their unmarried, divorced, or widowed counterparts in China’s public hospitals. The possible reason is that married acupuncture practitioners often have to balance clinical responsibilities with substantial family obligations, which can dilute professional focus and lead to greater fatigue and psychological strain. Thirdly, age was a key factor in acupuncture practitioners’ burnout, which is in line with earlier research (4). Our study further found that burnout levels tend to increase with acupuncture practitioners’ age in China’s public hospitals. The possible reason is that acupuncture practitioners highly depend on the long-term accumulation of acupuncture knowledge and clinical experience (44). Older practitioners, who typically possess more acupuncture skills and richer clinical experience, are often more trusted and favored by patients (41). This may result in higher work intensity and greater physical strain, contributing to persistent physical and mental exhaustion. Fourthly, years of work experience were a key factor in acupuncture practitioners’ burnout, which is in line with earlier research (4). Our study further found a general upward trend in acupuncture practitioners’ burnout with longer professional tenure in China’s public hospitals. The possible reason is that, over time, practitioners increasingly confront entrenched challenges. These obstacles weaken their professional identity and sense of accomplishment, thereby exacerbating burnout. Fifthly, the professional title was a key factor in acupuncture practitioners’ burnout, which is in line with earlier research [A (7)]. Our study further found that acupuncture practitioners holding intermediate titles experienced the highest levels of burnout in China’s public hospitals. The possible reason is that this group typically serves as the main clinical workforce, carrying heavy workloads while lacking sufficient authority, autonomy, and access to resources. Such conditions make them particularly prone to professional anxiety. Taken together, these findings suggest the importance of targeted interventions. Strategies such as physician-patient empathy training, family-friendly workplace policies, age-sensitive task redistribution, sustained support programs for experienced practitioners, and balanced workload and authority for those with intermediate titles may be critical to alleviating burnout among acupuncture practitioners in public hospitals.

Analysis of organizational factors reveals that institutional support for acupuncture departments was a key factor in acupuncture practitioners’ burnout, which aligns with earlier research (29). Our study further found that insufficient institutional recognition and support significantly increased acupuncture practitioners’ burnout. The possible reason is that, in China’s public hospitals, acupuncture is often regarded as a complementary therapy, making it difficult for practitioners to secure adequate organizational resources. Consequently, practitioners may feel their professional contributions are undervalued and their roles marginalized, reducing their confidence and engagement. Secondly, organizational management systems were a key factor in acupuncture practitioners’ burnout, which is in line with earlier research (45). Our study further found that rigid organizational management systems exacerbated acupuncture practitioners’ burnout (46). The possible reason is that the hierarchical and bureaucratic structures in public hospitals often limit practitioners’ work autonomy and constrain the flexible, individualized treatment approach fundamental to acupuncture. Such limitations may foster feelings of powerlessness and professional dissatisfaction among practitioners. Thirdly, remuneration was a key factor in acupuncture practitioners’ burnout, which is in line with earlier research (47). Our study further found that inadequate remuneration significantly increased burnout among acupuncture practitioners in China’s public hospitals. The possible reason is that acupuncture’s inherent characteristics of being “simple, effective, convenient, and inexpensive” (29), which keep treatment fees low. As a result, acupuncture practitioners typically receive lower incomes compared to their peers in other departments (45). This perceived imbalance between effort and reward fosters feelings of inequity and undermines practitioners’ sense of professional value. Fourthly, career advancement was a key factor in the burnout of acupuncture practitioners, which aligns with earlier research (48). Our study further found that restricted career advancement and limited career development significantly increased acupuncture practitioners’ burnout. The possible reason is that, in China’s public hospitals, the design of career advancement and development systems generally favors western medicine practitioners. As a result, acupuncture practitioners face career institutional disadvantages and stringent promotion criteria, which frequently result in professional frustration, emotional distress, and disengagement (49). Fifthly, departmental competition was a key factor in acupuncture practitioners’ burnout, which is in line with earlier research (50). Our study further found that intense competition between departments significantly increased acupuncture practitioners’ burnout (51). The possible reason is that, in China’s public hospitals, acupuncture departments are frequently positioned as secondary or supportive units, acupuncture practitioners often find themselves at a disadvantage in departmental competition. This can result in shrinkage of acupuncture departments, restricting practitioners’ professional development and weakening their sense of identity and belonging. Finally, interpersonal relationship within hospitals was a key factor in acupuncture practitioners’ burnout, which is in line with earlier research (33). Our study further found that complex interpersonal environments significantly increased acupuncture practitioners’ burnout. The possible reason is that, in China’s public hospitals, the performance-oriented management system often creates a zero-sum atmosphere. Acupuncture practitioners have to devote substantial emotional efforts to managing relationships with colleagues and supervisors, as well as resolving interpersonal conflicts, which often leads to exhaustion. Taken together, these findings recommend that it is of great value to enhance the strategic position of acupuncture departments in hospital, adopt a flexible management system centered on treatment efficacy, increase reimbursement for acupuncture services, create dedicated promotion pathways for acupuncture practitioners, implement differentiated departmental evaluations, and offer interpersonal skills training. These interventions may substantially reduce acupuncture practitioners’ burnout and enhance their professional well-being in public hospitals.

Analysis of occupational factors shows that, firstly, work intensity was a key factor in acupuncture practitioners’ burnout, which is in line with earlier research (52). Our study further found that high workloads and demanding schedules significantly increased acupuncture practitioners’ burnout (53). The possible reason is that, in China’s public hospitals, like other physicians, acupuncture practitioners frequently manage heavy patient loads, resulting in long workdays and insufficient rest (54). This not only leads to cumulative physical fatigue but also contributes to emotional exhaustion. Moreover, acupuncture requires continuous manual operation and sustained focus throughout each treatment session to ensure precision and safety. Maintaining this prolonged period of intense concentration often results in chronic mental fatigue and physical depletion. Additionally, prolonged standing or bending postures expose practitioners to occupational health risks such as muscle strain, cervical spine issues, and lower back disorders (55). Secondly, the work specialization was a key factor in acupuncture practitioners’ burnout, which is in line with earlier research (15). Our study further found that the demanding requirements for knowledge, experience, and technical skill in acupuncture place considerable pressure on practitioners (22). The possible reason is that, as an invasive therapeutic technique, acupuncture demands accurate diagnosis, careful point selection, proficient manipulation of needles, and flexible adjustments to treatment plans according to individual patient conditions. These complex demands impose constant pressure on practitioners’ substantial medical knowledge, extensive clinical experience, and advanced technical skills, leaving practitioners in a constant state of professional tension and anxiety. Furthermore, acupuncture theory is abstract and complex, with a vast body of knowledge to memorize and master. The heavy cognitive load of this learning process frequently results in mental fatigue and emotional burnout. Taken together, to alleviate excessive work intensity, public hospitals should scientifically optimize consultation schedules and patient loads, introduce mechanisms for flexible scheduling, rotational leave, and dynamic adjustment of workloads, and provide regular occupational health checkups. To address the pressures of work specialization, public hospitals should strengthen mentorship and clinical teaching systems, establish platforms for case discussion, experience sharing, and knowledge exchange, and provide designated learning time or professional development leave to ensure practitioners have adequate opportunities for growth.

Analysis of societal factors shows that, firstly, the patient negativity was a key factor in acupuncture practitioners’ burnout, which is in line with earlier research (30, 39). Our study further found that patients’ negative emotions—such as disrespect, provocation, suspicion, and verbal accusations—significantly intensified practitioners’ burnout. The possible reason is that, in China’s public hospitals, tense physician–patient relationships have resulted in diminished respect for physicians from patients (53, 56). To ensure treatment effectiveness, acupuncture practitioners are required to maintain patient responsibility, yet this sustained emotional labor often results in suppression and fatigue, eventually leading acupuncture practitioners to emotional detachment and depersonalization. Furthermore, due to the relatively long treatment duration and gradual onset of therapeutic effects in acupuncture (57), some patients may question or even criticize practitioners’ professional competence. Such a lack of recognition often generates frustration and irritability among acupuncture practitioners. In more severe cases, hostile or confrontational behavior from patients may lead acupuncture practitioners to feel unsafe, heightening the risk of doctor–patient conflict. Secondly, public misunderstanding about acupuncture was a key factor in acupuncture practitioners’ burnout, which is in line with earlier research (29). Our study further found that misunderstanding, skepticism, and prejudice toward acupuncture in the broader public further exacerbate practitioners’ burnout. The possible reason is that the lack of scientific and nuanced understanding of acupuncture among the public (29), which fuels misperceptions about acupuncture’s techniques, safety, and efficacy. In (58), misleading media reports, negative publicity, and the malpractice of unlicensed practitioners have undermined the reputation of the profession, deepening public mistrust and prejudice. In this context, acupuncture practitioners are deprived of essential social recognition and institutional support, leaving their professional value underestimated and their occupational identity marginalized. This erosion of recognition may foster negative emotions such as depression, frustration, and helplessness. Taken together, to address the patient negativity, public hospitals should provide regular training in emotional management, patient communication, and conflict resolution, while also strengthening institutional security systems to safeguard practitioners. At the same time, to address the public misunderstanding, efforts should be made to enhance the public promotion of acupuncture, establish mechanisms for positive opinion guidance, and elevate the entry standards for acupuncture practitioners to protect professional credibility.

This study has several limitations. First, although it identified multiple dimensions of factors influencing acupuncture practitioners’ burnout, it did not examine how these factors operate as an integrated system in shaping burnout. Future research could further explore the systemic mechanisms through which these factors jointly affect acupuncture practitioners’ well-being. Second, the study employed a cross-sectional design, with data largely derived from acupuncture practitioners’ self-perceptions shaped by a specific social context. This design may limit the ability to establish causal relationships among variables. Future studies could incorporate quasi-experimental or longitudinal designs to dynamically capture the long-term effects of multiple dimensions of factors on acupuncture practitioners’ burnout in public hospitals. Third, variations in cultural, institutional, and economic contexts may result in different patterns of burnout among acupuncture practitioners in other regions. As the sample in this study was drawn exclusively from China’s public hospitals, the generalizability of the findings may therefore be limited.

## 5 Conclusion

Acupuncture practitioners represent an essential professional group within China’s public hospitals. However, under the modern hospital management system, acupuncture practitioners have long found themselves in the awkward position of being ‘insiders as outsiders’. On one hand, they play an indispensable role in hospital operations, routinely providing long hours of intensive, high-quality clinical care, thus becoming the busy ‘insiders’ of the public hospital system. On the other hand, because acupuncture is often regarded as a ‘complementary’ therapy, these practitioners are commonly marginalized, receiving insufficient institutional backing and limited social recognition (59), thus becoming the marginalized ‘outsiders’ of the public hospital system. This ‘insiders as outsiders’ status significantly contributes to high levels of burnout among acupuncture practitioners and poses substantial challenges to their professional development.

Based on a nationwide survey, this study identified a moderate overall level of burnout among acupuncture practitioners in China’s public hospitals. Guided by the person-environment fit framework, our study revealed key factors influencing acupuncture practitioners’ burnout from four dimensions: individual, organization, occupation, and society. Key factors include individual factors (gender, marital status, age, years of work experience, professional title), organizational factors (institutional support, organizational management systems, remuneration, career advancement, departmental competition, interpersonal relationships), occupational factors (work intensity, work specialization), and societal factors (patient negativity, public misunderstanding).

Based on these findings, we propose a series of interventions. At the individual level, we recommended physician-patient empathy training, family-friendly workplace policies, age-sensitive task redistribution, sustained support programs for experienced practitioners, and balanced workload and authority for those with intermediate titles. At the organizational level, we recommended enhancing the strategic position of acupuncture departments in hospitals, adopting a flexible management system centered on treatment efficacy, increasing reimbursement for acupuncture services, creating dedicated promotion pathways for acupuncture practitioners, implementing differentiated departmental evaluations, and offering interpersonal skills training. At the occupational level, we recommended optimizing consultation schedules and patient loads, introducing mechanisms for flexible scheduling, rotational leave, and dynamic adjustment of workloads, providing regular occupational health checkups, strengthening mentorship and clinical teaching systems, establishing platforms for case discussion, experience sharing, and knowledge exchange, and providing designated learning time or professional development leave. At the societal level, we recommended providing regular training in emotional management, patient communication, and conflict resolution, strengthening institutional security systems to safeguard practitioners, enhancing the public promotion of acupuncture, establishing mechanisms for positive opinion guidance, and elevating the entry standards for acupuncture practitioners.

These findings enrich the broader literature on occupational health by highlighting the unique challenges faced by acupuncture practitioners, a group often absent from mainstream burnout research. Furthermore, these findings offer practical strategies for hospital administrators, policymakers, and health system leaders to optimize hospital management systems, improve acupuncture practitioners’ working conditions, and ultimately safeguard their well-being.

## Data Availability

The data supporting the conclusions of this article are available upon reasonable request from the corresponding author.
